# Limited congruence exhibited across microbial, meiofaunal and macrofaunal benthic assemblages in a heterogeneous coastal environment

**DOI:** 10.1038/s41598-018-33799-9

**Published:** 2018-10-19

**Authors:** Sorcha Cronin-O’Reilly, Joe D. Taylor, Ian Jermyn, A. Louise Allcock, Michael Cunliffe, Mark P. Johnson

**Affiliations:** 1 Ryan Institute & School of Natural Sciences, NUI Galway, University Road, Galway, H91 TK33 Ireland; 2 Marine Biological Association of the UK, The Laboratory, Citadel Hill, Plymouth PL1 2PB UK; 3 Centre for Sustainable Aquatic Ecosystems, Harry Butler Institute, Murdoch University, 90 South St, Murdoch, 6150 Western Australia Australia; 4 University of Salford, School of Environment & Life Sciences, Peel Building, Salford, M5 4WT UK; 5 Marine Biology and Ecology Research Centre, School of Biological and Marine Sciences, Plymouth University, Drake Circus, Plymouth PL4 8AA UK; 6 School of Veterinary and Life Sciences, Murdoch University, 90 South St, Murdoch, 6150 Western Australia Australia

## Abstract

One of the most common approaches for investigating the ecology of spatially complex environments is to examine a single biotic assemblage present, such as macroinvertebrates. Underlying this approach are assumptions that sampled and unsampled taxa respond similarly to environmental gradients and exhibit congruence across different sites. These assumptions were tested for five benthic groups of various sizes (archaea, bacteria, microbial eukaryotes/protists, meiofauna and macrofauna) in Plymouth Sound, a harbour with many different pollution sources. Sediments varied in granulometry, hydrocarbon and trace metal concentrations. Following variable reduction, canonical correspondence analysis did not identify any associations between sediment characteristics and assemblage composition of archaea or macrofauna. In contrast, variation in bacteria was associated with granulometry, trace metal variations and bioturbation (e.g. community bioturbation potential). Protists varied with granulometry, hydrocarbon and trace metal predictors. Meiofaunal variation was associated with hydrocarbon and bioturbation predictors. Taxon turnover between sites varied with only three out of 10 group pairs showing congruence (meiofauna-protists, meiofauna-macrofauna and protists-macrofauna). While our results support using eukaryotic taxa as proxies for others, the lack of congruence suggests caution should be applied to inferring wider indicator or functional interpretations from studies of a single biotic assemblage.

## Introduction

Taxa are typically thought to vary in their tolerance of environmental stresses and contaminants^1,2^. This is the basis of biotic indices like the AZTI Marine Biotic Index (AMBI) used to summarize ecological quality from the relative abundance of macroinvertebrates classified into groups representing different tolerance levels^2^. For marine sedimentary environments, measurements of ecological quality often involve macrofauna^3^. Other groups, such as bacteria, protists or archaea, are surveyed less frequently^4–6^. It is not clear to what extent the response of different groups to environmental gradients is congruent, i.e. respond in similar ways. If distributions of different groups lack congruence, the generality of indices derived from a single assemblage are restricted. Without congruence, more consideration must be given to choosing the most suitable biotic assemblage for the monitoring purpose and context (i.e. macrofauna *vs*. meiofauna^7^ or bacteria^8^, oil rig *vs*. coastal monitoring). In addition, variability in ecosystem function is unlikely to be predictable from a single assemblage^9^ if other functionally important groups respond in different ways to shared environmental conditions.

A lack of congruence has been observed previously, among sedimentary taxa^5,10^. Benthic archaea and bacteria have responded separately to shared environmental variables in the North Sea^5^ while nematode and microbial assemblages have shown non-congruent patterns in heavy-metal contaminated soils from an explosive manufacturing plant in Scotland^10^. However, some spatial variation in benthic assemblages has been found to be congruent across groups, for example between macrofauna and foraminifera around a sewage sludge disposal site in the Firth of Clyde^6^ or nematodes and macrofauna at a coastal site in Brazil^11^. Studies of similarity in response have often examined a clearly identified gradient of impact associated with oil spills or fish farm cages^12,13^ and also include manipulated increases in temperature^14^. Levels of contaminants and environmental conditions may, however, covary in complex ways. For example, different contaminants can come from spatially separated sources. In coastal environments, natural environmental conditions will vary with factors such as depth and wave exposure. In addition, anthropogenic contaminants like hydrocarbons and metals are commonplace stressors in these areas. This sort of multiple-stress context provides a more demanding test of congruence in taxon responses to environmental and anthropogenic stresses.

An example of a potentially complex seafloor with various sources of contaminants is provided by Plymouth Sound (Southwest England). The City of Plymouth has around 250,000 inhabitants and is home to a long-established naval dockyard. The Tamar and Plym rivers drain into the Sound and the catchment contains now-abandoned metal mines and there are reports of relatively high hydrocarbon levels^15^. We examined the congruence of five benthic groups (archaea, bacteria, microbial eukaryotes/protists, meiofauna and macrofauna) to multiple gradients in Plymouth Sound. Each group contributes to a series of key ecosystem services such as nutrient cycling, primary production and trophic transfer of energy^16,17^, but rarely are they considered together. Potential explanatory variables were sediment granulometry, hydrocarbon content, metal concentrations, nutrients and organic matter. The null hypothesis was that, in an environment with multiple gradients, there will be no congruence in the relationships among sites as expressed by the taxa present in different groups. As macrofauna can influence other groups by sediment reworking, an index of bioturbation (community bioturbation potential, BP_c_)^18^ and the distribution of different functional types of bioturbation were derived to test whether these variables influenced the occurrence of taxa within groups.

## Results

### Environmental and contamination gradients

**Figure 1:**
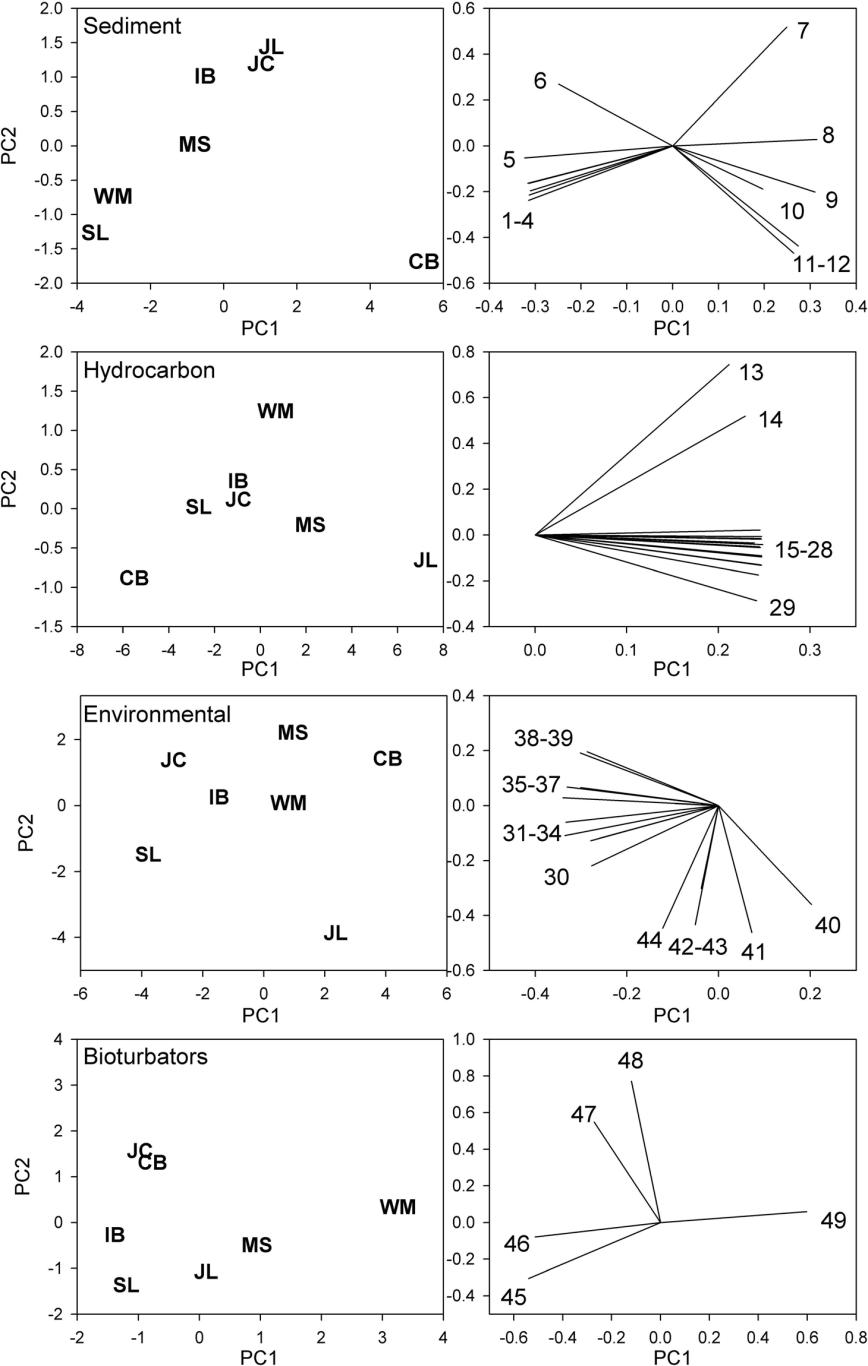
Principal components analysis of sediment, hydrocarbon, environmental and bioturbator variables across sites. Left hand panels show site scores used as predictor variables in subsequent canonical correspondence analysis. Right hand panels show loadings of variables on the first and second principal components. Site labels; CB) Cawsand Bay, IB) Inner Breakwater, JC) Jennycliff Bay, MS) Mallard Shoal, WM) West Mud, JL) St. John’s Lake, SL) Sutton Lock. Variable labels; 1) % medium silt, 2) fine silt, 3) coarse silt, 4) very fine silt, 5) clay, 6) very coarse silt, 7) very fine sand, 8) fine sand, 9) medium sand, 10) coarse sand, 11) very coarse sand, 12) very fine gravel, 13) acenaphthene, 14) phenanthrene, 15) fluorene, 16) pyrene, 17) benzo(b)fluoranthene, 18) benzo(g,h,i)perylene, 19) chrysene, 20) anthracene, 21) Total PAHs, 22) acenaphthylene, 23) benzo(k)fluoranthene, 24) indeno(1,2,3-cd)pyrene, 25) benzo(a)pyrene, 26) benz(a)anthracene, 27) dibenzo(a,h)anthracene, 28) fluoranthene, 29) naphthalene, 30) total N, 31) total P, 32) Zn, 33) Pb, 34) Cu, 35) Cd, 36) As, 37) S (SO_4_), 38) Cr, 39) Co, 40) C (CaCO_3_), 41) total C, 42) C (organic), 43) N (NO_3_), 44) Hg, 45) surficial modifiers, 46) biodiffusors, 47) downward conveyors, 48) upward conveyors and 49) upward/downward conveyors.

Principal components analysis (PCA) identified a number of different gradients across the sampled sites in Plymouth Sound (Fig. 1). PCA scores from different sets of variables were generally not correlated, implying that the data reduction process identified independent gradients across samples (mean pairwise correlation 0.02, no PCA score pairs significantly correlated). The main axis for granulometry variables separated sites across a spectrum of particle sizes (PC1 associated with 76% of the variation between sites, Supplementary Table 1). Cawsand Bay was the site with the highest sand particle fraction, with Sutton Lock dominated by silts. Hydrocarbons were generally positively correlated, resulting in a first principal component sorting sites by overall contamination level (95% of variance associated with PC1 hydrocarbon, Supplementary Table 2). The PCA of environmental variables contained a gradient stressing covariation in arsenic and a number of metals (Co, Cr, Cd, Cu, Zn and Pb). This identified Cawsand Bay as a less contaminated site and Sutton Lock as the most heavily contaminated. Surficial modifier and biodiffusor functional types were positively correlated and more associated with the coarser sediment sites, with upward/downward conveyors more common at West Mud.

### Phylogenetic and macrofaunal diversity

A total of 5,566 operational taxonomic units (OTU) were identified to various taxonomic levels as active constituents across archaea, bacteria, protists and meiofauna (1543, 2126, 1791 and 106 OTUs respectively) and total number of species/OTUs was calculated for each group (Supplementary Table 3). Broadly, archaeal rRNA transcript sequence libraries were characterised by representatives from three major phyla; Crenarchaeota, Euryarchaeota and Parvarchaeota. The novel uncharacterised archaeal order YLA114 was frequently detected in sequence libraries at all sites (relative abundance of 46–82%) except from Sutton Lock, where the rRNA transcript library was dominated by the Nitrosopumilales OTUs (74%). Bacterial rRNA transcript sequence libraries across all sites were dominated by the phyla Bacteriodetes, Firmicutes and Proteobacteria. Order Clostridiales (Firmicutes) was relatively abundant at Cawsand Bay (45%) and rare at all other sites (2–4%). Desulfobacteriales were largely present at all sites, relatively highest at Inner Breakwater (21%) and lowest at Cawsand Bay (8%). The Cyanobacteria order Croococcales was relatively abundant in the sequence libraries for Sutton Lock (15%) compared to all other sites (0–0.75%).

Most of the protists in the 18S rRNA transcript sequence libraries were Ochrophyta, specifically diatoms, except at Mallard Shoal where Ciliophora had a higher relative abundance (43%) in the rRNA transcript library compared to all other sites (0–8%). Foraminifera, class Monothalamea, were most abundant in the rRNA transcript library for Sutton Lock. Platyhelminthes and Nematoda made up a large fraction of meiofaunal reads in 18S rRNA transcript sequence libraries. Sequence reads for the Platyhelminthes were most abundant at St. John’s Lake, while Nematoda were dominant in sequence libraries at Jennycliff Bay (70%, 66% respectively). Nematoda and Copepoda are often classed as meiofauna. Our macrofaunal definition is consistent with standard, sieve-based benthic survey methods (organisms larger than 500 µm^19^). Both of these taxa were therefore recorded as part of the macrofaunal assemblage, with smaller nematodes in the meiofauna. The macrofaunal assemblage included 216 taxa mostly identified across two phyla, Annelida and Arthropoda (Supplementary Table 4). The polychaete family Cirratulidae were most abundant at Sutton lock and Inner Breakwater, but all other sites had Cossuridae (detritus feeding polychaetes) or Ampharetidae (bristle worms) as the most abundant taxa. The highest abundance of Arthropoda was found at Sutton Lock (19%) due to a large presence of Aoridae (amphipod) females. Pollution tolerant taxa (e.g. *Aphelochaeta marioni*) were more common at Sutton Lock.

The resulting Infaunal Quality Index (IQI)^3^ value for Sutton Lock was 0.83, as it was at St. Johns Lake. An ecosystem health assessment based on macrofauna would therefore rank Sutton Lock and St John’s Lake as the most impacted sites, although all sites lie above the standard threshold (0.75) used to describe ecosystem status as high (Supplementary Table 5).

### Congruence in assemblage structure

**Figure 2:**
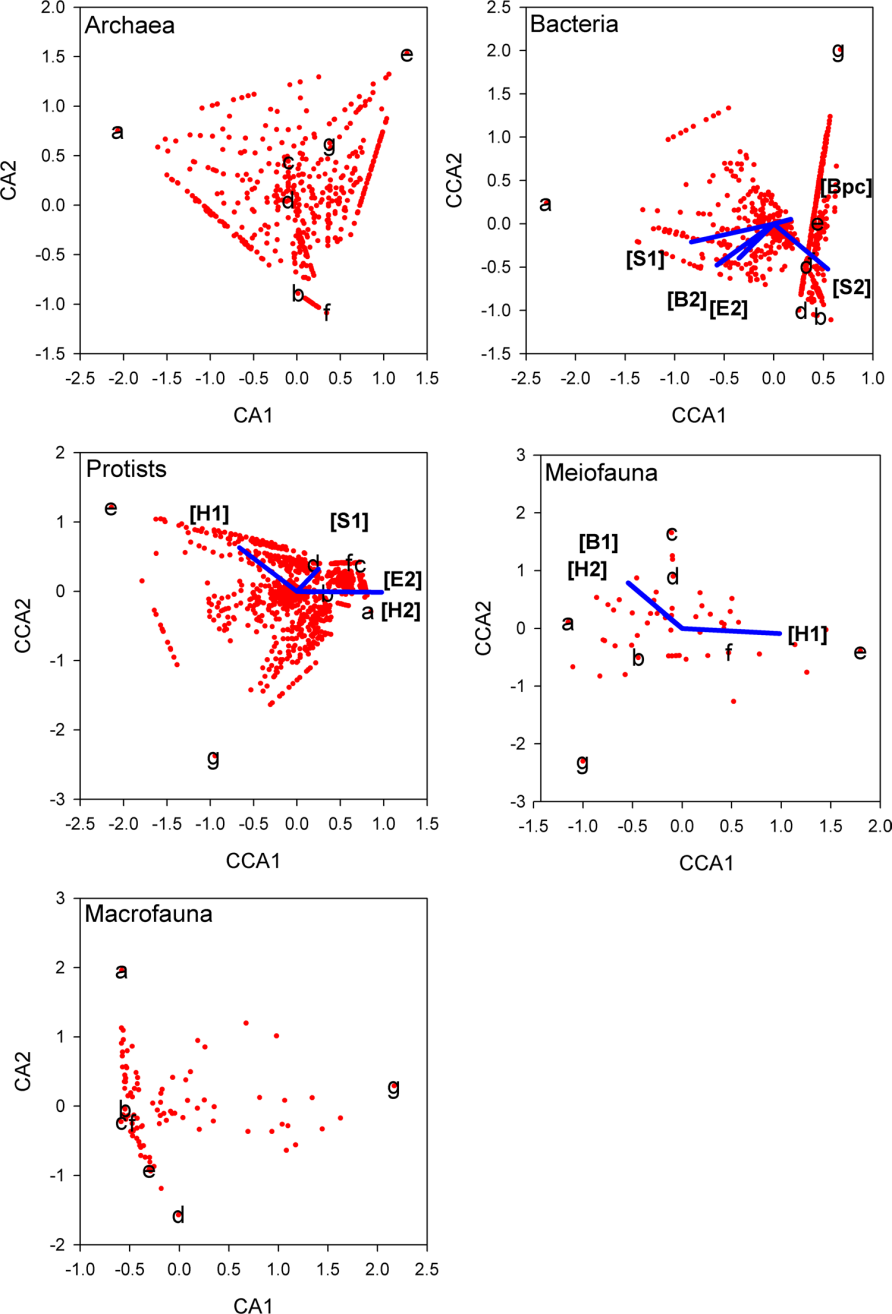
Correspondence analysis plot for each taxon group. Red points are the positions of individual taxa with respect to sites. Where predictor variables were significantly associated with pattern across the sites, these are added to the plots as lines, with direction and magnitude from the origin indicating the influence of the variable. Site labels; (**a**) Cawsand Bay, (**b**) Inner Breakwater, (**c**) Jennycliff Bay, (**d**) Mallard Shoal, (**e**) West Mud, (**f**) St. John’s Lake, (**g**) Sutton Lock. Variable labels; S1) sediment PC score on axis 1, S2) sediment PC2, H1) hydrocarbon PC1, H2) hydrocarbon PC2, E2) environmental PC2, B1) bioturbation PC1, B2) bioturbation PC2 and BP_c_) community bioturbation potential index.

Results from canonical correspondence analysis (CCA)^20^ differed across taxonomic groups with varying numbers of predictor variables significantly associated with inter-site variation in assemblage structure (Fig. 2). Archaeal and macrofaunal assemblages did not vary in response to gradients of any of the predictor variables examined. In contrast, sediment, environmental and bioturbation-related variables were associated with variation in the bacterial assemblage. Variation in both protist and meiofauna was related to hydrocarbon gradients, with additional roles for sediment structure and environmental variables in the former and bioturbation in the latter.

Although there appear to be some consistently outlying sites in the CCA plots, these patterns were not universal. Archaea, bacteria and macrofauna at Cawsand Bay were clearly separated from other sites, but the same site was not an outlier for meiofauna or protists. Out of 10 possible comparisons, only the PCO ordination pairs of meiofauna-protists, meiofauna-macrofauna and protist-macrofauna were congruent (p < 0.05, PROTEST randomisation tests). The average correlation between dissimilarity matrices for these congruent pairs was 0.631 (SE 0.097) compared to 0.141 (SE 0.098) for the other seven pairs.

## Discussion

The variations in assemblage structure across sites were not generally congruent across benthic groups: only three out of the 10 possible group pairs showed congruence in inter-site dissimilarities with congruence only evident between eukaryotic groups. Previous studies have investigated congruence among eukaryotes in the spatial patterns of macrofauna, meiofauna and protists. Nematode and macrofaunal samples have been seen to have congruent patterns of assemblage structure in Araçá Bay, Brazil^11^. The environmental gradients associated with glacial meltwater in a fjord were associated with congruence of foraminifera and macrofauna^21^.

A lack of congruence has been shown, however, when comparisons spread beyond eukaryotic groups. Similarities in response among benthic taxa were investigated between seven benthic sites in the North Sea and none of the groups investigated (bacteria, archaea, ammonia-oxidising bacteria and ammonia-oxidizing archaea) were congruent^5^. In a study of contaminated soils, relationships were found between soil chemistry and PCA-reduced microbial variables (such as plate counts, proportion of metal resistant bacteria and marker fatty acids), but not between univariate summaries of nematode diversity and the soil or microbial variables^10^. The current study is the first study to investigate all five major benthic groups simultaneously and it further supports an absence of congruence between eukaryotes and prokaryotes, or between archaea and bacteria alike.

The patterns observed in Plymouth Sound do not reflect separation into concordant and non-concordant groups by sampling or identification methods (core samples *vs*. subsample, morphological *vs*. molecular). Molecular *vs*. morphological approaches, although kept constant in some studies showing congruence^6,11^, are also constant in others where congruence is lacking^5^. Congruence in Plymouth Sound is evident between a morphologically identified group (macrofauna) and two molecularly defined groups (protists and meiofauna) whereas it is lacking between some of the molecularly defined pairs. This suggests that the results are not determined by differences in the methods used for group characterisation.

There are clearly differences between the evidence from molecular methods and direct counts. An important issue for measures of diversity and richness is that, when sequencing rRNA genes or transcripts from eukaryotic organisms, relative abundances of sequence reads often don’t relate directly to individuals. This is due to variation in rRNA copy numbers per cell/individual. Microeukaryotes such as ciliates may have many thousands of copies of rRNA genes within a single cell^22^ and with macrofauna and meiofauna, the size of the organism will also influence representation in sequence libraries^23^. Therefore, statistical tests using presence/absence data are advisable, as we have used here when examining congruence (PROTEST and Mantel tests). As RNA was used here, this could create a bias towards representation of the more active members of the community, reflecting higher transcript copy numbers in actively metabolizing cells^24^. In sedimentary systems, using RNA may be important to overcome the potential problem of relic DNA in sediment^25,26^ or the detection of inactive or resting cells that have settled from the water column onto the sediment. However, many studies have used RNA and DNA in assessing sedimentary microbial communities and found results to be largely comparable in terms of species richness^24,27^. Despite differences in methodology, several studies have shown that molecular techniques are comparable to direct counts for aquatic monitoring and ecological patterns are the same across pollution gradients^28,29^. In the assessment of meiofauna and macrofauna, it is important to note that while the 18S rRNA gene is wholly suitable for broad assessment of richness of taxa within benthic systems, the mitochondrial marker Cytochrome c oxidase I (COI) will give more accurate species-level taxonomic assignments, although this marker is limited by very few reference database sequences in comparison to the 18S rRNA gene^30^.

Molecular techniques are becoming more widely used in aquatic monitoring and biodiversity assessments and several detailed reviews have now been produced outlining the methods and limitations^31–34^. However, laboratory methods and bioinformatics analyses remain highly variable between studies, making cross comparison at the OTU level difficult. For example, different DNA and RNA extraction methodologies extract different taxonomic groups more efficiently, particularly for bacteria^35^, leading to sometimes very different patterns of OTU richness. Also, different bioinformatic pipelines can influence OTU richness^36^. The approaches we have taken in this study have been used across a number of other studies, but ultimately the community needs to develop standardized approaches for aquatic monitoring using molecular techniques.

There may be size-related aspects of how taxa respond to the perceived environmental gradients^37^, but these are not easily identified given the size differences between macrofauna and other groups and the lack of congruence between similar-sized taxa (i.e. archaea and bacteria). A lack of congruence may also reflect the divergence of stress responses in groups with different evolutionary histories^38^. In contrast, recent findings support similar responses to temperature increase across microbes and metazoans, irrespective of taxon size and phylogeny^14^. This may indicate that, when considering a dominant changing factor such as temperature, congruence may occur across divergent lineages.

Of course, the results from Plymouth Sound may be specific to the extent and type of gradients present. As anticipated, Plymouth Sound sediments showed a number of independent gradients of environmental contaminants. Responses to these gradients were identified for three (bacteria, protists and meiofauna) of the five groups studied and different combinations of variables were associated with changes in assemblage structure for each case. Congruence may emerge in situations where the environmental gradients are more aligned (e.g. synchronous increase/decrease of variables) and the range of each gradient is greater (e.g. around well-defined pollution sources) or where the spatial scale of the study is larger such that smoothing of small-scale variability with respect to larger regional patterns occurs^39^. Additional limitations may exist with the temporal setting of this study, as it represents one time point. The season of sampling may have increased the abundances of particular taxa, e.g. Cyanobacteria and the uncharacterised order YLA114 recognized to dominate during autumn^40^. It is unclear whether these patterns will remain constant through seasons as most assemblage structures vary temporally; for example through recruitment events linked to seasonal fluctuations in resources^41^.

Beyond the potential issues in assuming one group can act as a proxy for another, a lack of congruence has implications for interpreting the ecosystem function and health of benthic sediments on the basis of sampling a single assemblage. Based on rRNA sequencing of groups, the results show active groups responding to separate environmental gradients while displaying different degrees of taxon turnover between sites. As all groups impact ecosystem function, this therefore implies that interpretations of ecosystem function based on one group will potentially be compromised by patterns in another^42^. As of yet, any differences in archaea, bacteria, protist and meiofauna among sites are difficult to interpret as biodiversity ecosystem function (BEF) relationships are little studied for these groups^43^. It seems likely that differing patterns of biodiversity across taxa may add to the potential causes of context-dependency already identified in BEF relationships^18,44^.

Restrictions on generalization caused by a lack of congruence extend further when considering ecosystem monitoring through biotic indices (e.g. AMBI)^2^. The congruence between spatial turnover in taxa for meiofauna, macrofauna and protists supports the application of these groups as proxies for each other, provided the temporal setting of the monitoring programmes remains constant. It may be advisable, however, to include an index of the archaea/bacteria consortium, if such an index is available for the ecosystem (e.g. MC-IBI for rivers)^1^. The result of eukaryotic congruence extends to a functional index (BP_c_) derived from macrofauna^18^, which was associated with variation in bacterial composition. Alternative variables derived from the composition of bioturbator functional types were also associated with variation in bacteria and meiofauna. While these results emphasise that trait-based approaches can potentially define the links between taxa (including eukaryote/prokaryote interplay), the approach will need further research to establish the repeatability of such associations and the mechanisms of interaction.

## Material and Methods

### Study area and sediment sampling

**Figure 3:**
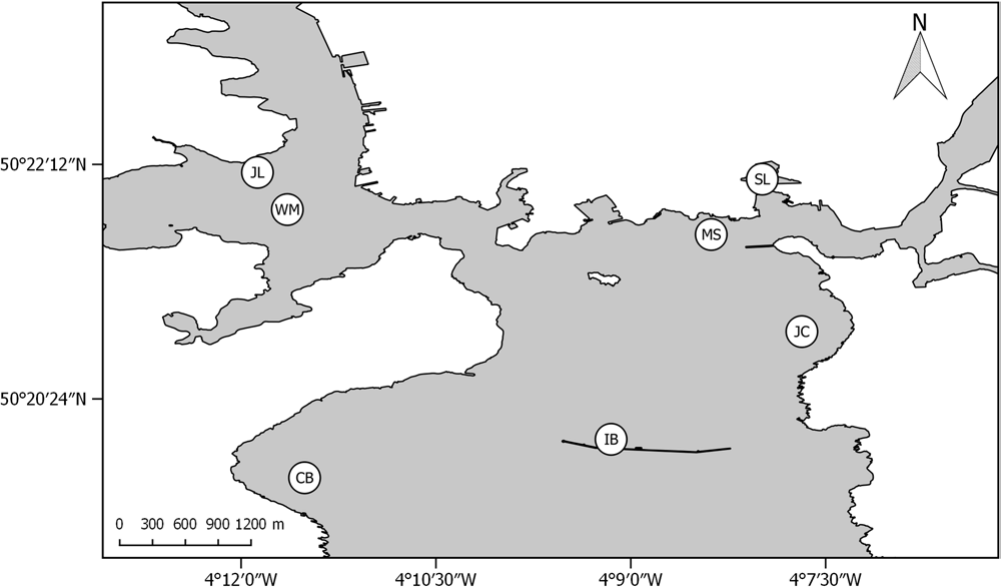
Plymouth Sound site map. Site labels; CB) Cawsand Bay, IB) Inner Breakwater, JC) Jennycliff Bay, MS) Mallard Shoal, WM) West Mud, JL) St. John’s Lake, SL) Sutton Lock. Map created using QGIS version 2.14 [QGIS Development Team (2016). QGIS Geographic Information System. http://qgis.org .

Benthic samples were taken using a HAPS corer over two days (12^th^-13^th^ of September 2013) at seven sites in Plymouth Sound (Fig. 3); Cawsand Bay (CB), Inner Breakwater (IB), Jennycliff Bay (JC), Mallard Shoal (MS), West Mud (WM), St. John’s Lake (JL) and Sutton Lock (SL). Three cores (15 cm diameter, 30 cm depth) were collected for measurement of environmental variables, microbial (i.e. archaea, bacteria and protists) and meiofaunal diversity analysis. Microbial and meiofaunal community analyses were based on three randomly spaced small cores (1 cm diameter, 5 cm depth) from each larger core and samples were placed into 1.5 mL centrifuge tubes and frozen immediately on dry ice for transfer to the laboratory where they were stored at −80 °C. A single replicate sample from each site was used for molecular analyses of these assemblages. A 200 g subsample was sliced from the top 5 cm layer of each larger core for hydrocarbon fingerprinting, trace metal and nutrient analysis. A single 100 g subsample from each site was used for granulometry and organic carbon content. Subsamples were stored in Rilsan® bags at −20 °C. Three additional cores (15 cm diameter, 30 cm depth) were taken for morphological characterisation of the macrofaunal community.

Laser particle sizing of sediments used nine 5 mg pseudo-samples of homogenised sediment samples from each site. Grain size distribution summary statistics were generated using GRADISTAT^45^. Organic content of samples were estimated using loss on ignition (LOI) where wet sediment samples were oven dried (24 hr at 100 °C) before being ignited in a muffle furnace (6 hr at 450 °C). The percentage weight loss after ignition was taken as a measure of total organic matter. Aliphatic and aromatic hydrocarbons were extracted in hexane/acetone and analysed using GC-FID. Polycyclic aromatic hydrocarbons (PAHs) were extracted in hexane/acetone/triethylamine and analysed using GC-MS^46^. Metals were extracted in nitric acid/hydrochloric acid using microwave digestions and analysed using ICP-OES^47^. Nutrients (nitrate, nitrite, phosphate) were measured using an autoanalyser^48^.

### Sediment RNA extraction, sequencing and bioinformatics

To mitigate some of the potential issues of relic DNA^49^ within sediments, RNA was used for microbial and meiofaunal community assessment. RNA is thought to give better representation of the “active” fraction of the communities, although this is a subject of debate^50^.

Sediment cores for microbial and meiofaunal analysis were homogenized and weighed (0.25 g) into tubes containing 0.5 g glass beads (100–300 μm, MPBIO) and 1 mL TRI Reagent® (Ambion) before bead beating. Samples were heated at 60 °C for 10 min before 600 μL of supernatant was transferred to a new tube containing 100 µl 1-bromo-3-chloro-propane and vortexed. The tubes were centrifuged to separate the organic and aqueous phases before the aqueous phase was transferred to QIA-shredder columns (Qiagen, U.K) containing 0.2 g polyvinylpolypyrrolidone to remove humic acids and phenolic compounds. The resulting filtrate was precipitated with 2-propanol (equal volume) and sodium acetate (1/10 volume) for 1 hr at −20 °C before the RNA pellet was isolated and washed by centrifugation. The RNA pellet was resuspended in 100 μL RNAse-free water and further cleaned using the RNeasy kit (Qiagen) according to the manufacturer’s instructions. DNase treatment was performed using RQ1 RNase-Free DNase (Promega) according to the manufacturer’s instructions. Control PCRs confirmed the presence of RNA only. Generation of cDNA was performed using an Omniscript RT kit (Qiagen) in accordance with manufacturer’s instructions.

Bacterial 16S rRNA, archaeal 16S rRNA and eukaryote 18S rRNA gene amplicon sequencing was carried as previously described^46^. Sequencing of amplified bacterial 16S rRNA and eukaryote 18S rRNA genes was performed on an Ion Torrent PGM (Life technologies) and sequencing of amplified archaeal 16S rRNA genes was performed on a MiSeq (Illumina) according to manufacturer’s instructions. Sequences were analysed using the QIIME version 1.8.1 software package and USEARCH version 8. Quality filters were used to remove short (<150 bp) and low quality reads (average Phred score < 25). Chimeras were then identified using UCHIME^51^ using the Greengenes database (release 13_5)^52^ for 16S and the SILVA database (version 128)^53^ for 18S as references. OTUs were clustered using the UPARSE algorithm^54^ defined at 97% similarity for 16S and 18S libraries. For the 18S libraries this similarity threshold was selected as it has been shown to be appropriate for a broad range micro-eukaryote taxonomic groups and specific groups such the Chlorophyta^55–57^. This cut-off has been shown to be appropriate for both macro/meio-organisms on mock communities^58,59^. However, it should be noted for other groups 99% (such as some groups of ciliates) may be more appropriate^60,61^.

Sequences were classified against their respective databases using UCLUST^62^ and OTU tables generated. Eukaryote sequence reads were split into protists using a proposed classification^43^ and meiofauna based on their taxon identification (e.g. Nematodes, Platyhelminthes, Ostracods and Rotifers). The OTU tables were rarefied to fixed sequence depth for each sample (Bacteria 8000, Archaea 4000, Protists 13000, Meiofauna 1000) and these OTU tables served as input for further statistical tests.

### Macrofaunal processing and community analyses

Macrofaunal samples were washed with water over a 500 µm sieve to remove excess sediment. Retained faunal samples were sorted, identified to species level, where possible, and enumerated using a combination of dissecting and compound microscopy. Specimens were dyed to ease identification when necessary using methylene blue (C_16_H_18_ClN_3_S, CAS: 61-73-4). Identified taxa were labelled and stored in a 70% Industrial Methylated Spirits solution inside glass snap-cap vials. Compilation of the species list was finalised by cross-reference and synonym adjustment consistent with the World Register of Marine Species index (WoRMS; http://www.marinespecies.org/index.php). To calculate the BP_c_ index for each site as described^18^, macrofaunal data was averaged across site replicates and macrofaunal wet weights were recorded following blot-drying for <5 seconds. The IQI^3^ incorporating the AMBI^2^ was estimated for macrofauna data to gauge ecosystem health, following HAPS to GRAB instrument conversion of volumes.

### Variable reduction and model fitting

The 49 variables measured at each site were reduced to a limited number of potential predictors of assemblage structure using PCA. Variables were grouped into sets reflecting sediment granulometry, hydrocarbon content, a more generic ‘environmental’ category (representing trace metals and other elements, nutrients and carbon) and a categorization of bioturbation functional types and BP_c_. The classification of bioturbators followed the sediment reworking functional types given for taxa^18^: surficial modifiers, biodiffusors, upward conveyors, downward conveyors and upward/downward conveyors. Macrofauna were assigned to functional types and relative frequencies of types were used in subsequent PCA.

Benthic assemblages were tested for association against the variable sets summarized in PCA scores using CCA^20^. CCA plots simultaneously show the dispersion between sites and species based on tables of sites by species, but also express the main axes as linear combinations of predictor variables. Even with variable reduction using PCA, the resultant set of nine predictor variables (eight sets of PCA scores using PC1 and PC2 for each grouped variable set and BP_c_ values) was sufficient to saturate the CCA for seven sites. The most informative predictors for ordinations were therefore chosen using forward stepwise selection. At each step, the most informative model was chosen on the basis of a pseudo-F statistic that compares the relative magnitude of the inertia (variance) around the constrained model with predictor(s) to the unconstrained model (no predictors). Significance of the pseudo-F statistic can be assessed by randomly permuting the data with the expectation that an informative predictor will have a lower inertia than the values obtained when there is no pattern in the data. Model building continued by adding predictors one at a time, stopping when additional predictors did not produce a significant reduction in inertia. In some cases, no individual variables provided a significant correlate of observed variation. In these cases, the CCA reverts to an unconstrained analysis, i.e. a correspondence analysis (CA). All ordinations and significance testing were carried out in the vegan package in R^63^.

The CCA analyses can identify important covariates of variation in taxon identity across sites. The congruence in spatial pattern among taxa can be examined using differences between sites based on taxon turnover (beta diversity) measures, quantified using the presence/absence based Jaccard dissimilarity. Jaccard dissimilarities can be interpreted as the probability that two taxa, drawn from separate sites, will not be shared between the sites^64^. If the different groups of taxa are structured in the same way among sites, dissimilarities between pairs of sites will be related. For example, sites that are relatively similar when measured in terms of archaeal taxa will also share more bacterial taxa than average. This hypothesis can be tested with a Procrustes test, where the minimum difference between ordinations following scaling and rotation is compared to the results of permuting random site composition (the PROTEST statistic)^65^. Ordinations based on Jaccard dissimilarities were made using principal coordinates analysis (PCO) in R. Procrustes statistics were based on all six dimensions from a PCO, as the first two axes only explained approximately 60% of the variation between sites. The Procrustes test is thought to have equal or better power than a Mantel test of the matrix of dissimilarities from one group correlated against dissimilarities from another group^65^. The Mantel test randomises one matrix to establish whether the correlation between a pair of dissimilarity matrices is greater than is likely to have occurred by chance. An advantage of a Mantel correlation is that it is easier to interpret than the PROTEST statistic, where the latter is only equivalent to a correlation when two-dimensional ordinations are superimposed. Mantel statistics (Spearman’s rank correlation) are therefore also presented to aid interpretation. The results of Mantel tests and PROTEST were identical in terms of statistical significance.

## Electronic supplementary material

Supplementary information

## Data Availability

The data generated and analysed during this study is available as supplementary information to this publication and molecular sequence data can be found at the European Nucleotide Archive (Accession reference code PRJEB24674). Taxon by site tables (.txt format) can be made available on request.
