# Supplementary material for: Limited congruence exhibited across microbial, meiofaunal and macrofaunal benthic assemblages in a heterogeneous coastal environment
Source: Sci Rep. 2018 Oct 19;8:15500. doi: 10.1038/s41598-018-33799-9 (PMC6195585; doi:10.1038/s41598-018-33799-9)
Supplement: Supplementary file 1 — Supplementary information [file 41598_2018_33799_MOESM1_ESM.pdf]

Limited congruence exhibited across microbial, meiofaunal and macrofaunal benthic assemblages in a heterogeneous coastal environment

Sorcha Cronin-O'Reilly[1,3,6]*, Joe D. Taylor[2,4], Ian Jermyn[1], Louise Allcock[1], Michael Cunliffe[2,5] & Mark P. Johnson[1]

Supplementary Information

RNA sequences: European Nucleotide Archive accession PRJEB24674

Supplementary Table 1: Fractions of sediment size particle analysis expressed as percentage (%) contribution. Textural groups listed as MuS: Muddy sand, and SMu: Sandy mud. Site labels; CB) Cawsand Bay, IB) Inner Breakwater, JC) Jennycliff Bay, MS) Mallard Shoal, WM) West Mud, JL) St. John's Lake, SL) Sutton Lock.

Site		CB	IB	JC	MS	WM	JL	SL
Textural Group		MuS	SMu	MuS	SMu	SMu	MuS	SMu
V. Coarse Sand	%	1	0	0	0	0	0.1	0
Coarse Sand	%	3.9	1.7	3.8	2.8	1.1	1.1	2.1
Medium Sand	%	16.1	7.2	9.4	8	4.2	7.1	5
Fine Sand	%	38.6	16.3	18.2	15.2	10.3	30.8	6.1
V. Fine Sand	%	22.9	21.3	25.9	18.1	13.3	27.4	10.1
V. Coarse Silt	%	4.1	16.3	14.6	13.5	12.6	7.7	14.4
Coarse silt	%	4.3	13	7.9	12	15	6.1	18
Medium Silt	%	4.4	0	8.6	13	18.9	7.6	19.5
Fine Silt	%	3.1	7.8	6.7	10	15.3	6.8	14.9
Clay	%	0.4	2	1.9	2.5	3.2	2	3.7

Supplementary Table 2: Environmental composition of sediments at investigated sites. All values are averages of three measurements. Some nutrients recorded as percentages (%), metals and other nutrients recorded in milligram per kilogram (mg/kg) and hydrocarbon levels given in micrograms per kilogram (µg/kg). Site labels; CB) Cawsand Bay, IB) Inner Breakwater, JC) Jennycliff Bay, MS) Mallard Shoal, WM) West Mud, JL) St. John's Lake, SL) Sutton Lock.

Parameter	Unit	CB	IB	JC	MS	WM	JL	SL
Total N	%	0.06	0.19	0.19	0.12	0.15	0.17	0.19
Total C		3.23	3.46	2.91	2.12	3.73	8.54	4.56
C(Org)		0.87	2.77	2.49	1.57	2.25	5.13	2.88
C(CaCO3)		2.37	0.7	0.42	0.55	1.48	3.41	1.68
N(NO3)	mg/k	0.64	0.69	0.42	0.75	0.7	0.87	1.08
N(NO2)	g	<1	<1	<1	<1	<1	<1	<1
P(PO4)		<0.3	<0.3	<0.3	<0.3	<0.3	<0.3	<0.3
As		9	31	38	22	24	16	31
Cd		0.03	0.17	0.31	0.13	0.11	0.07	0.31
Cr		29	38	40	39	36	25	43
Cu		18	78	119	42	52	38	163
Fe		2038	2627	2929	27447	23999	1901	3287
Hg		0.04	0.52	0.45	0.09	0.6	1.15	1.03
Ni		24.59	26.4	28.62	28.94	25.263	20.73	33.93
Pb		23	79	103	39	56	53	123
Zn		67	151	196	118	113	91	618
Acenaphthene	µg/kg	<8	25	25	29	39	19	38
Acenaphthylene		<12	50	49	106	76	19	152
Anthracene		23	105	105	131	163	65	332
Benz(a)anthracene		85	372	410	579	434	277	981
Benzo(a)pyrene		77	428	501	826	599	281	1280
Benzo(b)fluoranthene		81	444	223	848	650	297	1180
Benzo(g,h,i)perylene		57	267	283	520	384	178	710
Benzo(k)fluoranthene		34	215	226	386	255	143	520
Chrysene		62	351	375	488	394	255	800
Dibenzo(a,h)anthracen		<23	64	71	127	86	45	192
Fluoranthene		133	651	629	900	619	519	1540

Fluorene	<10	28	31	38.5	40	19	76
Indeno(1,2,3-	47	220	241	440	318	151	654
Naphthalene	<9	48	50	76	41	27	138
Phenanthrene	53	335	296	366	397	226	488
Pyrene	117	590	603	881	821	477	1590
Total Aliphatics >C5-35	2690	5830	5160	21000	11900	4560	5350
Total Aromatics >C5-35	<100	5000	5240	14000	70800	7080	3630

Supplementary Table 3: Total species or operational taxonomic units/OTUs (S) for all groups at each site. Site labels; CB) Cawsand Bay, IB) Inner Breakwater, JC) Jennycliff Bay, MS) Mallard Shoal, WM) West Mud, JL) St. John's Lake, SL) Sutton Lock.

Diversity index	Group	CB	IB	JC	MS	WM	JL	SL
Total species/OTUs (S)	Archaea	374	348	110	840	234	411	112
	Bacteria	706	417	769	701	461	535	803
	Protists	688	592	940	600	702	623	576
	Meiofauna	34	35	31	19	26	40	16
	Macrofauna	76	78	79	53	61	40	78

Supplementary Table 4: Macrofaunal abundance matrix displaying recorded abundances of 216 taxa identified. Records presented as decimals due to averaging abundances across three replicate cores. Site labels; CB) Cawsand Bay, IB) Inner Breakwater, JC) Jennycliff Bay, MS) Mallard Shoal, WM) West Mud, JL) St. John's Lake, SL) Sutton Lock.

Taxon	CB	IB	JC	MS	WM	JL	SL
Acanthicolepis asperrima	0	0	0	0	0	0	0.33
Acholoe squamosa	0	0	0.33	0	0	0	0
Ampharetidae	0	0	0	0	0	0.33	0
Ampharete acutifrons	0	0	0	0	0.33	1.33	0
Ampharete	0.33	0.33	0.33	0	0	0	0
Ampharete finmarchica	3.67	2.33	2.67	2	0.33	0.33	0.33
Aphelochaeta marioni	0	0	0	0	0	0	162.33
Myrianida brachycephala	0	0	0	0	0.33	0	0
Myrianida inermis	0	0	0.33	0	0	0	0
Myrianida	0	0	0.67	0	0.67	0	0
Brania pusilla	0	0	0	0	0	0	0.67
Capitella	0.67	1.33	3.33	0.5	0	2	5

Caulleriella alata	0	0	0	0	0	0	2
Caulleriella bioculata	0	0	0	0	0	0	7.67
Caulleriella viridis	0	0	0	0	0	0	3
Caulleriella	0	0	0	0	1	0	1
Chaetozone zetlandica	0	0	0	0	1.33	0	0
Chaetozone gibber	12	91.67	27.33	27.5	10	11	6.67
Chaetozone setosa	1.33	22	51.67	18.5	6	36.67	0
Cirratulus	0	0	0	0	0	0	2
Cirriformia tentaculata	0	0	0	0	0.67	0	10.67
Clymenura	0.33	3	10.33	0.5	1	0	0
Cossura longocirrata	0	14	3.33	3.5	1	50.33	0
Diplocirrus glaucus	0	0.67	0	0	0	0	0
Eteone barbata	0	0	0.33	0	0	0	0
Euclymene oerstedii	7.67	3.33	26.33	3.5	0	0	0
Euclymene	4.33	1.33	0	0	0.33	0	0
Eumida bahusiensis	0.33	0.67	2.33	1	1	0	0.33
Exogone	0.67	0	0	0	0	0	0
Parexogone hebes	1.33	4.33	11.33	2.5	0	0	0.67
Exogone naidina	2.33	0.33	0	0	0	0	0.33
Galathowenia oculata	0	0	0	13	0	2.33	0
Glycera	0.33	0	0	0	0	0	0
Heteromastus filiformis	0	0	1.33	0.5	0	0.33	0
Lumbrineris	0	0	0	1	0	0	0
Lumbrineris futilis	0	0.33	0	0	0	0	0
Lumbrineris coccinea	0	0	0	0	0	0	0.33
Hilbigneris gracilis	0	0	0	0	0	0	0.33
Lumbrineris tetraura	0.33	1.33	1.67	0.5	0	0	0
Magelona alleni	0.33	0	1.67	0.5	1.33	0.33	0
Magelona equilamellae	0	0	0	0	0	0	0.33
Magelona filiformis	14.33	0.33	1.33	1.5	0.33	1	0.33
Magelona minuta	6	10	12.67	18.5	0	0.67	0
Magelona	0.33	0	0	0	0	0	0
Maldanidae	0	0.33	2	1.5	0	0	0
Malacoceros	0	0	0	0	0.33	0	0
Malacoceros fuliginosus	0	0.67	0.67	0	0	0.33	0
Malacoceros tetracerus	1	0.33	0	0	0	0	0.33
Malmgrenia andreapolis	0	0	0.33	0	0	0	0
Malmgrenia marphysae	0	0	0.33	0	0	0	0
Marphysa bellii	0	0.67	0	0	0.33	0	0
Marphysa	0	0.33	0.33	0	0	0	0
Mediomastus fragilis	0.67	2	0	0	8.33	1.33	9.33
Melinna palmata	13	46	36.67	88.5	100.33	19	2.67
Kirkegaardia dorsobranchialis	9.67	36	27.67	7	8.33	0.33	1
Neoamphitrite affinis	0.33	0	0	0	0	0	0
Lysidice unicornis	0	0	0	0	0.67	0	0

Nephtys caeca	0.33	0	0	0	0	0	0
Nephtys hombergii	0.67	0.33	1.67	2	1.33	2.67	0.33
Nephtys kersivalensis	0	0	0	0	0.33	0	0
Nephtys paradoxa	0	0	0.33	0	0.33	0	0
Nephtys	0	0	0	0	0.67	0	0
Nereis	0	0	0	0	0	0	0.33
Notomastus	1	0.33	3.33	0.5	1.33	0.33	0.67
Owenia fusiformis	1.33	1.33	0.33	0.5	0	0	0
Paradoneis eliasoni	0	0	0	0	0	0	0.33
Paradoneis lyra	0	0	0	0.5	0	0	0
Paraonides	0	1	0	0.5	0	0	0
Parapionosyllis minuta	0	0	0	0	0.33	0	0.33
Amphictene auricoma	0	0	0	0	0.33	0	0
Lagis koreni	1	0	0.33	0	0.33	0	0
Phyllodoce groenlandica	0	0	0.33	0	0	0	0
Phyllodoce mucosa	0	0	0	0	0	0	2.33
Podarkeopsis capensis	0.33	0	1	0.5	0	0	0.33
Poecilochaetus serpens	0	0	0	0	0.33	0	0
Polycirrus	0	0.33	0	0	0	0	0
Dipolydora coeca	0	0	0	0	0	0.33	0.67
Polydora	0	0	0	0	0.67	0	0
Polynoidae	0.33	0.33	0	0	0	0	0
Praxillella affinis	0	0.67	0	0	0	0	0
Praxillella gracilis	0.33	0	0	0	0	0	0
Prionospio cirrifera	0	0	0.33	0	0	0	0
Prionospio multibranchiata	0	2	3.33	0.5	0	0	0
Protodorvillea kefersteini	0	0	0	0	0	0	0.33
Pseudomystides limbata	0	0	0	0	0	0	0.33
Pseudopolydora paucibranchiata	0.33	0	0	0	29.67	1.33	38.33
Pseudopolydora pulchra	0	0	0	2	0.33	0.33	0
Pygospio elegans	0	0	0.33	0	0.67	7.33	0
Sabellidae	0.33	0	0	0	0	1	0
Scalibregma inflatum	0	0.33	0	1.5	0.33	0	0
Sigalion squamosus	0.33	0	0	0	0	0	0
Spio filicornis	0.33	0.33	0.33	0	0	0	0.33
Spio	3	0.67	3.33	17	5	0.33	3
Spionidae	0	0.33	1.33	0	1.67	0.33	0
Spiophanes bombyx	0	0.33	0	0	0	0	0
Sternaspis scutata	0	11.33	0	1.5	0	1.67	0
Streblospio shrubsolii	0	0	0.33	0	0	10.33	0
Fimbriosthenelais zetlandica	0	0	0	0	0.33	0	0
Syllidia armata	0	0	0	0	0	0	0.33
Terebellides stroemii	0	0.67	0	0	0	0	0
Oligochaeta	0.33	0	0	0	0	0	0
Tubificoides amplivasatus	0	15.67	12.67	12	0.67	0	0

Tubificoides benedii	0	0	0	0	0.33	0	27.33
Tubificoides galiciensis	0	14.67	11.33	1	1	15.33	35.67
Tubificoides heterochaetus	0	0.33	0	0.5	0	0	0.33
Tubificoides insularis	0	0	0	0	0	0	0.33
Tubificoides	0	0.33	0	0	0	0	0
Tubificoides pseudogaster	0	0	0	0.5	0	0	11.67
Tubificoides swirencoides	0	10	1	0	0	0.33	0.67
Websterinereis glauca	0	0	0	0	0.33	0	0
Ampelisca brevicornis	0.67	0.33	1	0	2.67	1	0
Ampelisca tenuicornis	2.33	2.67	2.67	12.5	4.67	0.33	0.33
Ampelisca typica	0.33	0	0	0	0	0	0
Ampelisca	0	0.33	0.33	0	0	0	0
Ampeliscidae	0	0	0	0	0	0.33	0
Amphilochoides boecki	0	0.33	0	0	0	0	0
Amphilochidae	0	0.33	0.67	0	0	0	0
Anoplodactylus virescens	0	0	1	0	0	0	0
Anoplodactylus petiolatus	0	0.33	0	0	0.67	0	0
Aora typica	0	0	0	0	0	0	7
Aoridae	0	0	0.33	0	1.67	0.33	43.67
Apherusa cirrus	0	0.33	0	0	0	0	0
Apseudopsis latreillii	0	0	0	0	0	0	31.67
Cheirocratus sundevallii	0.33	0	0	0.5	0	0	0
Copepoda	1	0	0	0	0.33	0.33	0.33
Corophium	0	0.33	0	0	0	0	0.33
Crassicorophium crassicorne	0	0	0	0	0	0	3
Chelicorophium curvispinum	0	0	0	0	0.33	0	0
Cressa dubia	0	0	0	0	0	0	0.67
Decapoda	0	0.33	0.33	0	0	0	0
Dexamine	0	0	0	0	0	0	0.33
Dexamine spinosa	0.33	0	0	0	0	0	1
Diastylis lucifera	0	0	0.33	0	0	0	0
Ericthonius punctatus	0	0	1.67	0	0	0	0
Ericthonius difformis	0	0	1.33	0	0	0	0
Eudorella truncatula	0	6.33	9.67	4	2.67	0	0
Gammaridae	0.33	0	0	0	0	0	0
Gammarus	0	0.33	0	0	0.67	0.33	0
Harpinia antennaria	0.33	0	0	0	0	0	0
Harpinia crenulata	0	0.67	0	0	0	0	0
Harpinia laevis	0	0	0	0.5	0	0	0
Harpinia serrata	2.33	0	0.33	0	0	0	0
Harpinia pectinata	0	0.33	0	0	0	0	0
Heterotanais oerstedii	0	0	0	0	0	0	0.33
Isaea montagui	0.33	0	0	0	0	0	0
Isopoda	0	0	0	0	0	0	0.33
Leucothoe incisa	1	0.33	0	2	0	0	0
Leucothoe lilljeborgi	0	0.33	0	0	0	0	0

Leptocheirus pectinatus	0	0	0	0	0	0	2
Chondrochelia savignyi	0	0	0	0	0	0	1
Liocarcinus navigator	0	0	0	0	0	0	0.33
Macropipus tuberculatus	0	0	0	0	0.33	0	0
Maera grossimana	0.67	0	0	0	0	0	0.33
Melita	0	0	0	0	0	0	1.67
Melita dentata	0	0.33	0	0	0	0	0
Abludomelita obtusata	0	0	0	0	0	0	0.33
Melitidae	0	0	0.33	0	0	0	0
Uromunna petiti	0	0	0	0	0	0	0.33
Ostracoda	0	0	0	0	0.33	0	0
Pagurus pubescens	0	0	0.33	0	0	0	0
Pariambus typicus	0.67	6.33	0.67	0	0	0	0.33
Perioculodes longimanus	2.67	0	2	0	0	0	0
Pseudocuma (Pseudocuma) simile	0.67	0	0	0	0	0	0
Photis longicaudata	0	0	0	0.5	0	0	0
Phoxichilidium femoratum	0	0	0.67	0	0	0	0
Phtisica marina	0	0	0	0	0	0	3
Siphonoecetes (Centraloecetes) kroyeranus	0	0	0	0	0	0	0.67
Stenothoe monoculoides	0	0	0.33	0	0	0	1.67
Metopa rubrovittata	0	0	0.67	0	0	0	0
Synchelidium haplocheles	0	0	0.33	0	0	0	0
Tanaidacea	0	0	0	0	0	0	0.33
Tanaissus lilljeborgi	0	0	0.33	0	0	0	1.67
Acrocnida brachiata	0.33	0	0	0	0	0	0
Amphiura	0.33	0	0	0	0	0	0
Amphiura filiformis	1	0.33	0	0	0	0	0
Asterina	0	0	0	0	0	0	0.33
Asteroidea	0	0	0	0	0	0	0.33
Echinocardium cordatum	1	0	0	0	0	0	0
Oestergrenia digitata	0	0	0.67	0	0	0	0
Ophiocten affinis	0	0	0.33	0	0	0	0
Ophiurida	2.33	0.33	1	0	0	0	0
Nematoda	0	0	0	1	2.67	0	60
Nemertea	0.67	0.67	0.33	1.5	0	0	0
Phoronis	1	2	2.33	2.5	1.67	0.33	0
Golfingia	0.67	0	0	0	0	0	0
Sipuncula	0	0	0	0	0.67	0	0
Abra alba	2.67	4.67	5.33	0	0.67	0	0
Abra nitida	2.33	0.67	0	0	0.33	0	0
Abra prismatica	0	0.33	0	0	0	0	0
Calyptraea chinensis	0	0	0	0	0	0	0.67
Cardioidea	0	0	0	0.5	0	0.33	0
Cephalaspidea	0.33	0	0	0	0	0	0

Chamelea gallina	1	0	0	0	0	0	0
Chamelea striatula	0.33	0	0	0	0	0	0
Mimachlamys varia	0.33	0	0	0	0	0	0
Parthenina interstincta	0	0	0	0	0	0	0.33
Clausinella fasciata	2.33	0	0	0	0	0.33	0
Cylichna cylindracea	0	0	0	0.5	0	0.67	0
Corbula gibba	1	1.67	0	0.5	0	0	0
Kurtiella bidentata	5	0	0.33	6.5	0.33	0	0
Lutraria angustior	0	0	0.33	0	0	0	0
Lucinoma borealis	0.67	0	0.33	1	0	0.33	0
Mactra stultorum	0.33	0	0	0	0.67	0	0
Mytilus	0	0	0	0	0.33	0	0
Nucula hanleyi	2.67	0.67	0.33	0	0	0	0
Parvicardium pinnulatum	0	0	0	0	0.33	0	0.67
Phaxas pellucidus	1.33	0.33	0.33	3	0	0	0
Philine aperta	0	0.33	0	0	0	0	0
Raphitoma linearis	0	0	0.33	0	0	0	0
Tellimya ferruginosa	3.33	0	0	0	0	0	0
Thracia phaseolina	0.67	0	0	0	0	0	0
Thyasira flexuosa	1.67	2.67	2	1	0	0	0
Turritella communis	0	0.33	3	1.5	0	0	0
Actinia	0	0.67	0.33	0	0	0	0
Edwardsia	0	3	0	2.5	0	0	0.67
Ascidiacea	0	0	0	0	0	0	1

Supplementary Table 5: Infaunal Quality Index output indicating ecosystem health of sites based on macrofaunal communities present. EG: Ecological group. Site labels; CB) Cawsand Bay, IB) Inner Breakwater, JC) Jennycliff Bay, MS) Mallard Shoal, WM) West Mud, JL) St. John’s Lake, SL) Sutton Lock.

Site	EG I (%)	EG II (%)	EG III (%)	EG IV (%)	EG V (%)	Most abundant taxa (% of sample)	IQI score	Ecological status
CB	38.2	14	26.6	20.4	0.8	Magelona filiformis (10.4%)	1.19	High
IB	8.6	4.2	25	49.4	12.8	Chaetozone gibber (26.8%)	0.9	High
JC	14.3	10.3	24.4	40.5	10.5	Chaetozone setosa (16.7%)	1	High
MS	17.6	4.5	50.2	22.3	5.4	Melinna palmata (32.5%)	0.97	High
WM	8.2	3.1	59.1	28.7	1	Melinna palmata (47.7%)	0.92	High
JL	4.5	1.9	24.5	58.6	10.5	Cossura longocirrata (29.2%)	0.83	High
SL	11.9	1.6	24.3	46.1	16.1	Aphelochaeta marioni (31.9%)	0.83	High
